# Optic flow based spatial vision in insects

**DOI:** 10.1007/s00359-022-01610-w

**Published:** 2023-01-07

**Authors:** Martin Egelhaaf

**Affiliations:** Neurobiology and Center for Cognitive Interaction Technology (CITEC), Bielefeld University, Universitätsstraße 25, 33615 Bielefeld, Germany

**Keywords:** Spatial vision, Motion detection, Optic flow, Behavioural control

## Abstract

The optic flow, i.e., the displacement of retinal images of objects in the environment induced by self-motion, is an important source of spatial information, especially for fast-flying insects. Spatial information over a wide range of distances, from the animal's immediate surroundings over several hundred metres to kilometres, is necessary for mediating behaviours, such as landing manoeuvres, collision avoidance in spatially complex environments, learning environmental object constellations and path integration in spatial navigation. To facilitate the processing of spatial information, the complexity of the optic flow is often reduced by active vision strategies. These result in translations and rotations being largely separated by a saccadic flight and gaze mode. Only the translational components of the optic flow contain spatial information. In the first step of optic flow processing, an array of local motion detectors provides a retinotopic spatial proximity map of the environment. This local motion information is then processed in parallel neural pathways in a task-specific manner and used to control the different components of spatial behaviour. A particular challenge here is that the distance information extracted from the optic flow does not represent the distances unambiguously, but these are scaled by the animal’s speed of locomotion. Possible ways of coping with this ambiguity are discussed.

## Behaviour needs to be orchestrated on a variety of spatial scales

The behaviour of moving animals takes place in space, in the case of flying animals, like most insects, in three-dimensional space. The range of distances relevant for spatial behaviour varies widely: For example, if a prey object is to be grasped, it must be within the grasping range of the animal’s limbs. Often, however, the goal object must first be reached by locomotion over a certain distance. On the path to such a goal, other objects may be in the way. These must then be identified as obstacles to be able to initiate suitable evasive manoeuvres. Objects that are obstacles under certain conditions can, however, serve as landing objects in other behavioural contexts. For both collision avoidance and landing decisions, the behaviourally relevant distance range is determined by the locomotion velocity as well as the time the animal needs to initiate an avoidance manoeuvre or a landing approach. However, if goals such as a prolific food source or the nest are to be found after a long excursion, the behaviourally relevant distance range may extend far beyond the spatial range that can be directly perceived by the animal. The wide range over which spatial information must be gathered and represented in the animal's brain implies a variety of underlying mechanisms.

Spatial vision is often taken to be essentially equivalent to stereoscopic vision, which is based on disparities between the retinal images of the two eyes. Stereoscopic vision is of central importance for primates in the near range when grasping and manipulating objects with their hands (Howard and Rogers 2012; Read 2021), but also for other animals such as toads aiming for a prey with their tong (Collett 1977) or praying mantids sitting in ambush to catch prey with the claws of their forelegs (Rossel 1983; Nityananda et al. 2018; O'Keeffe et al. 2022). However, for geometric reasons, stereoscopic vision is not practicable at distances much larger than the immediate grasping range, regardless of the species. Especially during fast locomotion in complex environments behavioural decisions need to be made often already at relatively large distances from objects. Then other sources of distance information are needed. Although a number of spatial information sources are available (Collett and Harkness 1982; Howard 2012), for moving animals ranging from insects to birds and humans, the optic flow (OF), i.e., the pattern of image shifts on the eyes induced during locomotion, plays a dominant role in guiding spatial behaviour at all distance ranges, from very close to far away.

## Optic flow as a source of distance information

OF has its basis in the image displacements on the eyes induced during self-motion of the animal. For geometric reasons, the OF pattern can be formally decomposed into two components, reflecting the translation vector and the corresponding rotation vector of self-motion in three-dimensional space. The rotational components of the OF pattern depend only on the rotation velocity of the animal, whereas the translational OF components are determined by both the translation velocity of the animal and the distance to the objects in the environment. The spatial information is thus contained exclusively in the translational OF component, which, at a given translation velocity, causes closer objects to move faster on the eyes than more distant ones. However, the spatial information is closely intertwined with the animal's translation velocity, as both a smaller distance to surrounding objects and a larger translation velocity lead to a larger image speed and thus a stronger OF. Hence the spatial information in the translational OF component is ambiguous (Longuet-Higgins and Prazdny 1980; Heeger and Jepson 1992). This important property of OF-based spatial information has consequences for spatial behaviour in various functional contexts.

Apart from this geometric ambiguity, it is important to note that OF information can only be detected by visual mechanisms if the retinal input signals are modulated in a time-dependent manner. This is not the case when the animal passes, for instance, a homogeneous, unstructured surface. Contrasts, such as object edges, where the brightness of the background differs from that of the objects, are therefore a prerequisite for determining OF and thus the spatial information based on it.

## Reduction of optic flow complexity by active vision strategies to facilitate spatial vision

**Fig. 1:**
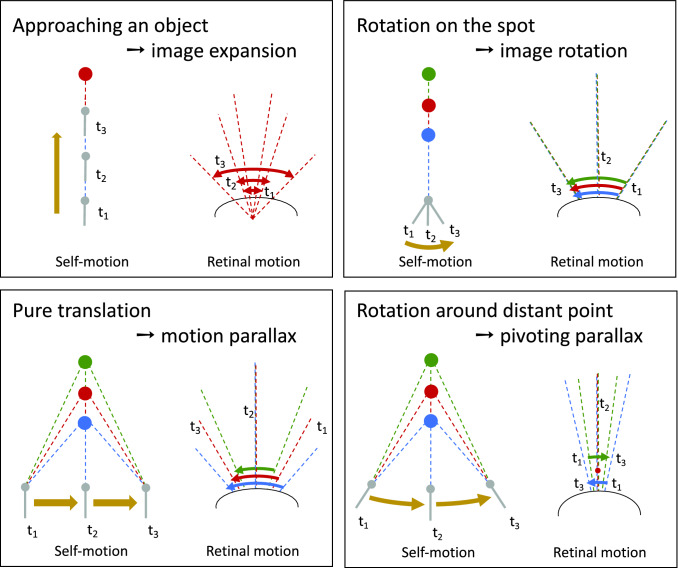
Optic flow induced by different types of self-motion in specific environments. Schematic representation of an animal's self-movement relative to three objects at different distance (left diagram in each box) and the corresponding OF (right diagram in each box) from a top perspective. The left diagrams show the position of the animal at three different times (*t*_1_, *t*_2_, *t*_3_) and the angle relative to the longitudinal axis of the body at which the objects are seen. The arrows in the right diagrams indicate the retinal image shifts of the objects induced by three types of self-motion and the corresponding OF at the respective time points. When approaching an object translationally, the object appears to become larger and larger as the distance decreases (image expansion); with pure rotation, the objects shift with the same angular velocity regardless of distance. If the animal translates e.g., sideways past objects of different distances, the corresponding retinal image speed depends on the distance, with the closer object moving faster than the more distant ones; this kind of motion parallax thus provides distance information relative to the animal. A rotation of the animal around a distant point (pivoting point) in the environment (here the red object) corresponds to a combined rotational and translational movement in body coordinates; as a consequence, the pivoting point does not move at all, while the near and far object move on the retina in different directions; the OF associated with such a pivoting parallax thus contains distance information relative to the pivoting point, rather than relative to the animal

As already mentioned, the spatial information contained in the OF pattern is interwoven in an intricate way with the actual movement information. Theoretically, under certain conditions, the translational OF component and, in the next step, the spatial information can be extracted computationally from this complex flow field (Longuet-Higgins and Prazdny 1980; Strübbe et al. 2015). However, the necessary computational processes are complex and would represent a great challenge for the small brains of insects. One way to reduce the computational effort of the nervous system for extracting behaviourally relevant spatial information from the OF is to actively shape the rotational and translational components of self-motion. Three such active vision strategies relative to environmental objects are particularly relevant in the context of spatial behaviour of insects; they are contrasted in Fig. 1 with a pure rotation of the animal around its vertical axis, which leads to OF without spatial information.

1. *Looming OF induced by translational locomotion towards an object:* The retinal image of an object that is approached enlarges more and more with a characteristic retinal speed that depends on the speed of self-motion and the distance to the object. Such looming stimuli are thus accompanied by a characteristic OF pattern that scales with the locomotion speed and contains distance information, which can be used for behavioural control in various contexts.
2. *Motion parallax induced by pure translational locomotion*: During pure translational locomotion, the projected image of an object on the retina shifts at a larger speed the closer it is to the animal. Hence, the distance to an object is defined at a given translation velocity of the animal by the object’s retinal velocity. For geometrical reasons, the OF is zero directly in the direction of translation (‘focus of expansion’) and increases in an equidistant environment within the visual field towards the direction orthogonal to the direction of translation where it is largest. Hence, an animal should move sideways, if distance information relative to an object needs to be gathered in the frontal visual field. Indeed, prominent sideways flight components can be observed in many behavioral situations when the animal appears to scrutinize the spatial layout of its environment.
3. *Pivoting parallax induced by a specific combination of translational and rotational locomotion*: Distance information relative to a behaviourally relevant goal location in the nearby environment can be generated by a specific combination of translational and rotational self-motion that leads to a rotation of the animal about this goal location, i.e., the pivoting point, rather than about the animal’s centre. For this peculiar form of locomotion, the OF scales for a given velocity of the animal with increasing distance to the pivoting point.

**Fig. 2:**
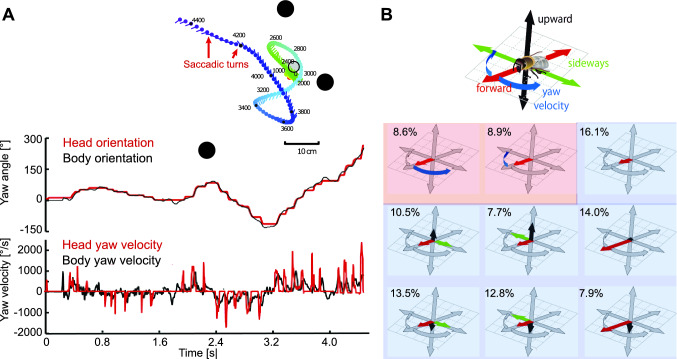
Saccadic flight and gaze strategy. **A** Inset: Trajectory of a flight of a bumblebee as seen from above after leaving an inconspicuous feeder placed between three textured cylinders (black objects). Each dot and line indicate the position of the bee in space and the viewing direction of its head at time intervals of 20 ms, respectively. The time is colour-coded (given in ms after the start of the flight at the feeder). Upper diagram: Yaw orientation of longitudinal axis of body (black line) and head (red line) of the flight trajectory shown in the inset. Note that step-like, i.e., saccadic direction changes, are more pronounced for the head than for the body. Bottom diagram: Yaw velocity of body (black line) and head (red line) of the same flight (Data from Mertes et al. 2014; Boeddeker et al. 2015). **B** Translational and rotational prototypical movements of honeybees during cruising and local landmark navigation in a flight arena. Flight sequences while the bee was searching for a visually inconspicuous feeder located between three cylindrical landmarks can be decomposed into nine prototypical movements using clustering algorithms. Each movement prototype is depicted in a coordinate system as explained by the inset. The lengths of the arrows indicate the size of the corresponding velocity component. Percentage values provide the relative occurrence of each prototype. More than 80% of flight-time correspond to a set of translational prototypical movements (light blue background) and only less than 20% have a systematic non-zero rotational velocity corresponding to the saccades (light red background) (Data from Braun et al. 2012)

Animals can use such active vision strategies in specific behavioural situations and environmental contexts (see below) to be able to capture behaviourally relevant spatial information as computationally parsimonious as possible. Apart from such specific situations, insects, at least those where this has been systematically studied, i.e., mainly flies and Hymenoptera, employ an active flight and gaze strategy even if their behaviour is not explicitly directed by objects. This saccadic flight and gaze strategy is characterized by a sequence of often extremely fast rotations, the saccades, and by intervals in which the animals primarily translate with a largely constant gaze direction, leading to an OF on the eyes in which the translational component dominates (Fig. 2A) (Land 1999; van Hateren and Schilstra 1999; Dickinson 2005; Zeil et al. 2008; Egelhaaf et al. 2012; Boeddeker et al. 2015; Kress et al. 2015; Muijres et al. 2015; Doussot et al. 2020). The relative duration of the intersaccades, i.e., the time during which the direction of gaze changes only slightly, accounts for more than 80% of the total flight time. Intersaccadic flight can be further subdivided into prototypical movements, each characterized by the specific relative contribution of forward and sideways flight components (Fig. 2B) (Braun et al. 2012). The saccadic flight and gaze strategy facilitates the evaluation of spatial information from the OF by largely separating the translational and rotational OF components behaviourally. This saccadic flight style is prevalent during most of the time that the insects are flying around, even if no immediate reference object for their behaviour is apparent. This makes sense because an animal should have constant access to spatial information about its immediate surroundings. Only then can the animal detect objects of whatever spatial dimension as existing and react appropriately to them—potentially using one of the dedicated active vision strategies mentioned above. In other words, the general saccadic flight and gaze strategy helps to segment a cluttered spatial scenery without much computational effort into nearby and distant objects and on this basis allows the animal to act towards those objects that are of special behavioural relevance by specific dedicated flight manoeuvres.

## Spatial behaviour based on optic flow information in insects

So far, it has been outlined how the OF can be simplified by specifically orchestrated locomotion in such a way that extraction of behaviourally relevant spatial information by the brain is possible with computationally parsimonious mechanisms; computational parsimony with simultaneous efficiency can be expected in particular from insects with their small brains. In the following, it is outlined how insects solve certain spatial behavioural tasks and what role OF might play as a source of spatial information in each case. The outlined behavioural tasks take place on a broad spectrum of spatial scales, which implies a broad spectrum of mechanisms for the acquisition of spatial information and behavioural control. The choice of behavioural tasks summarised covers only a small spectrum of the tasks insects face and is limited to those that have each been studied to date at least to some extent.

### Peering movements for behaviour in the near range

Even though stereoscopic vision is used at least by praying mantises in the near range to strike prey, i.e., in a situation where they should be as motionless as possible to not scare away the potential prey (Rossel 1983; Nityananda et al. 2016; Read 2021), there are situations where sitting insects use distance information based on OF to control their behaviour. For instance, sitting locusts generate translational sideways movements with their bodies (‘peering’), which lead to motion parallax on their eyes, especially in the frontal visual field. The resulting distance information is then used to jump accurately to a twig or another target object (Collett 1978; Kral and Poteser 1997; Kral 2012). In experiments in which the parallax velocity of the object and thus its apparent distance were systematically manipulated by the experimenter during the animal's peering movements, it could be shown that the locust’s jump velocity is controlled by OF-based distance information (Sobel 1990). If the animals are sitting, peering movements of the body and head are the only way to gain distance information through movement parallax. Peering movements also play a role during flight in a variety of behavioural contexts, such as when the insect pinpoints the distance to a food source location (Boeddeker and Hemmi 2010), assesses its flight altitude (Baird et al. 2021) or the width of a gap to be flown through (see below).

### Regulating flight speed depending on the spatial layout of the environment

**Fig. 3:**
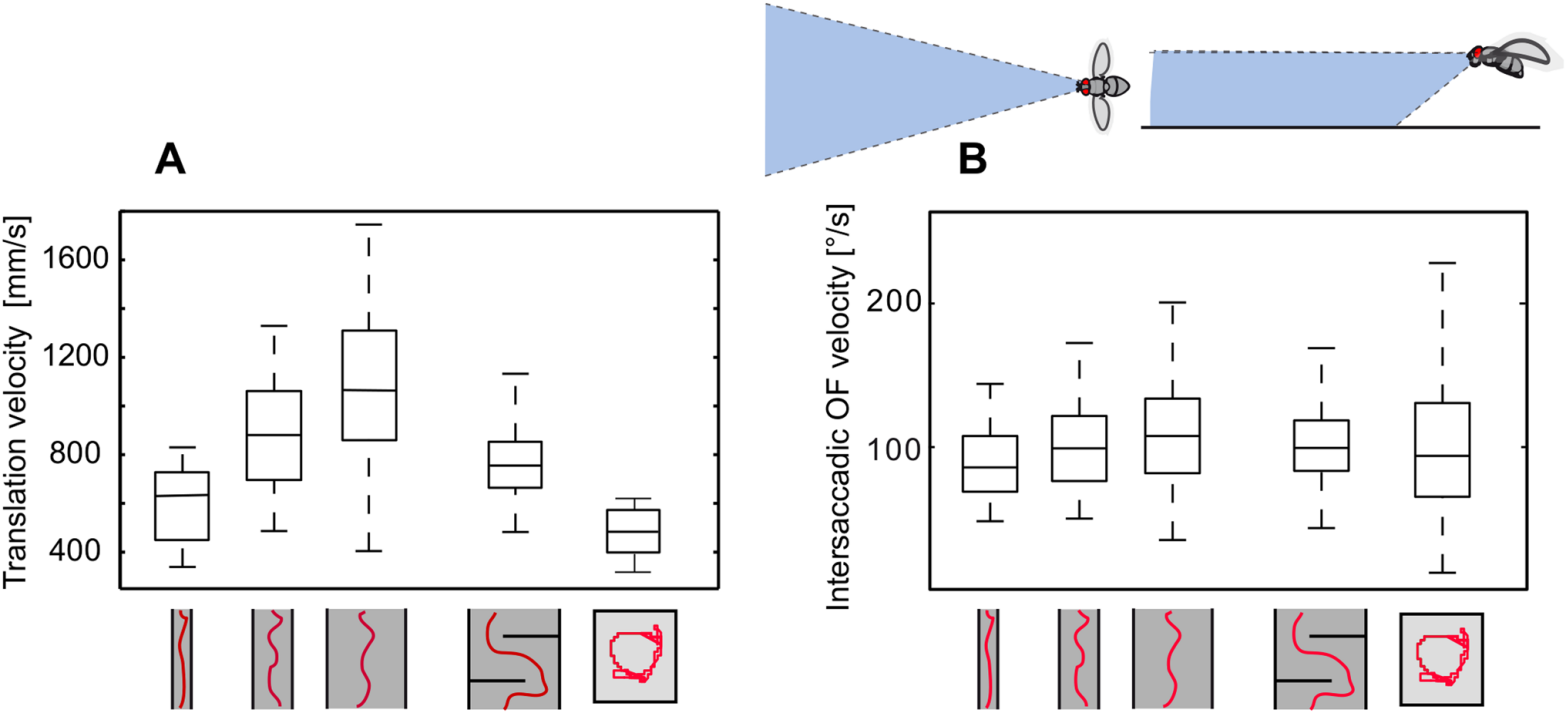
Flight speed controlled by spatial layout of the environment. **A** Control of translational velocity in free-flying blowflies in environments with different spatial characteristics. Boxplots of the translational velocity in flight tunnels of different widths, in a flight arena with two obstacles and in a cubic flight arena (sketched below data). Translation velocity strongly depends on the geometry of the flight arena. **B** Boxplots of the retinal image velocities within intersaccadic intervals experienced in the fronto-ventral visual field (see sketches above data diagram) in the different flight arenas. In this part of the visual field, the intersaccadic retinal velocities are kept roughly constant by regulating the translation velocity according to clearance with respect to environmental structures. The upper and lower margins of the boxes in **A** and **B** indicate the 75th and 25th percentiles, and the whiskers the data range (data from Kern et al. 2012)

Flying insects regulate their overall translation velocity depending on the spatial layout of the environment. For instance, insects decelerate if the width of a flight corridor becomes narrower or if the flight path is partially obstructed by objects (Srinivasan et al. 1996; Baird and Dacke 2012; Kern et al. 2012; Serres and Ruffier 2017). Flight speed is thought to be controlled by OF generated during translational flight. Flies, bees, and moths were concluded to keep the OF on their eyes at a “preset” level by adjusting their flight speed. Accordingly, they decelerate when the translational OF increases, for instance, while passing a narrow segment of a flight corridor (Srinivasan et al. 1991; Baird et al. 2005; Portelli et al. 2011; Kern et al. 2012; Linander et al. 2015, 2016; Stöckl et al. 2019; Grittner et al. 2021). Not all parts of the visual field contribute equally to the input of the flight velocity controller: For flies the most prominent role can be attributed to the intersaccadic OF generated in eye regions looking in front of the insect (Fig. 3). In these regions of the visual field, the intersaccadic retinal velocities are kept in a range where the responses of the motion vision system still increase monotonically with increasing velocity and decrease with decreasing velocity (see below; Egelhaaf et al. 2012; Kern et al. 2012). Bumblebees have been concluded to rely on the OF and thus on relative nearness information in the lateral visual field when negotiating narrow corridors, but on ventral OF and, thus, relative nearness information to the ground in wider terrain; the optic flow in the frontal field of view plays a particularly important role when it comes to detecting and responding to changes in the proximity of the environment well before these changes in the spatial layout are encountered (Baird et al. 2010; Linander et al. 2015, 2016).

Although it is clear overall that flight speed is reduced in confined and cluttered terrain, there are still unanswered questions and sometimes conflicting findings about the underlying control mechanisms. It is generally accepted that the OF is balanced between the two eyes for a wide range of environmental conditions, as is reflected in experimental studies in flight tunnels on a variety of insect species meandering along the tunnel’s midline ("centering response"). However, if the textural and/or spatial properties of the environment in front of the two eyes differ in certain ways deviations from centering can be observed (Srinivasan et al. 1991; Serres et al. 2008; Dyhr and Higgins 2010; Baird and Dacke 2012; Kern et al. 2012; Linander et al. 2015, 2016; Chakravarthi et al. 2017; Serres and Ruffier 2017; Lecoeur et al. 2019; Baird 2020). Two findings in particular show that balancing the OF in front of the two eyes is not always essential to allow insects to pursue a roughly straight flight course: (1) Functionally blinding one of the eyes hardly affects the ability of flies to fly straight, though monocular flies tend to fly slower and to tilt their body axis slightly to the side of the seeing eye. Detailed analyses of the OF patterns on the non-occluded eye show that this surprising performance of monocular flies can be explained by a kind of monocular optomotor equilibrium, i.e., the superimposed translational and rotational OF components on the seeing eye sum to zero (Kern and Egelhaaf 2000). (2) Large OF asymmetries on the two eyes may, however, also occur under more natural conditions, for instance, when an insect flies along a textured wall with an open field on the other side of a flight tunnel after having the bee trained to fixate and fly towards a feeder close to the wall at the other end of the tunnel. This situation, that has been experimentally analyzed for bees, cannot be accounted for by balancing OF across the two eyes. Instead, a lateral OF regulator has been proposed, i.e., a feedback system that is aimed at keeping the unilateral OF constant (Serres et al. 2006, 2008).

### Landing

The ability to land is essential for all flying animals and thus represents a central spatial vision requirement. As the animals are in motion when initiating landing manoeuvres, OF is predestined to be used as a source of spatial information. Successful landings require continuous precise adjustment of flight speed, body orientation and leg posture to the respective distance from the landing site to ensure a smooth landing. Usually, the animals decelerate before landing to ensure a smooth touchdown (see below). However, some hymenopteran species were found to do the opposite and accelerate just before landing; not much is known about the potential role of OF in controlling this peculiar behaviour (Shackleton et al. 2019; Tichit et al. 2020). Two fundamentally different landing situations can be distinguished, namely landing on relatively flat surfaces and landing on small objects. In both spatial situations, OF is considered the decisive source of information for the control of the landing behaviour.

When landing on a flat horizontal surface, honeybees were concluded to pursue a computationally simple OF-based strategy by keeping the retinal velocity of the ground constant during the approach, thus automatically ensuring that flight speed is close to zero at touchdown (Srinivasan et al. 2001). If the surface they approach is vertical, the bees also gradually and automatically reduce their flight speed by keeping the speed of the image expansion constant (Baird et al. 2013). Whereas honeybees continuously decelerate when landing on a vertical surface, bumblebees were concluded to employ a somewhat different strategy by exhibiting a series of deceleration bouts. During each bout, the bumblebee keeps the relative rate of expansion constant; from one bout to the next, the bumblebee tends to shift to a higher constant relative rate of expansion. This landing strategy is interpreted to be faster than that described for honeybees (Goyal et al. 2021).

Under natural conditions, insects often land on objects that differ from their background in some way. They can land even on objects that are indistinguishable from the background in texture and colour and can only be detected based on relative motion cues (Lehrer et al. 1988; Srinivasan et al. 1989; Kimmerle et al. 1996; Kern et al. 1997). Regardless of the characteristics that distinguish a landing object, the OF induced during the landing approach with its strong looming component plays the decisive role in controlling the pre-landing deceleration and the timely extension of the legs before touchdown; this has been shown in both free flight and tethered flight experiments (Wagner 1982; Borst 1990; Kern et al. 1997).

### Collision avoidance

**Fig. 4:**
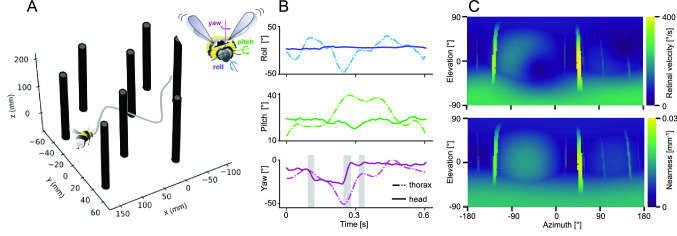
Nearness to obstacles indicated by retinal optic flow during intersaccadic intervals. **A** Schematic of a sample flight trajectory of a bumblebee in a flight tunnel with cylindrical obstacles. The inset indicates the three components of rotational movement. **B** Time resolved roll, pitch and yaw orientation of the bee’s thorax and head in world coordinates for the flight shown in **A**. Whereas the roll and pitch angles of the body change tremendously during the flight manoeuvres, the corresponding head angles stay relatively constant due to rapid head–body coordination. However, the yaw angle of both body and head need to change to allow the bee to fly around the obstacles; whereas the yaw orientation of the head changes rapidly in a saccadic fashion and only much less during intersaccadic intervals, the changes of body orientation are more sluggish. **C** Upper diagram: Sample snapshot of the total OF for the full spherical field of view at a given instant of time during an intersaccadic interval based on the head trajectory and orientation. Bottom diagram: Snapshot of the nearness map for the head trajectory and orientation at the same instant of time. The spatial map of intersaccadic OF generated from the head data closely reflects the nearness map that represents the geometric profile of the environment with the nearby obstacles being more salient in the OF map than the more distant ones (Data from Ravi et al. 2022)

An existential challenge to spatial vision, especially for fast-moving animals, is avoiding collisions with objects that are in the way. OF is the most relevant sensory source of information for solving this task. It could be shown that the OF during flights of bumblebees in a tunnel with numerous obstacles represents the proximity to these; this is a consequence of the saccadic flight and gaze strategy and the associated relative stability of the gaze direction during the intersaccadic intervals (Fig. 4) (Ravi et al. 2022). Obstacle avoidance behaviour was concluded to be essentially robust over the entire brightness range under which bumblebees are naturally active (Baird 2020). How insects deal with the OF characteristics under different collision avoidance conditions has been analyzed in detail during both tethered and free flight. The evasive reactions in free flight are based on the extremely fast saccadic rotations characteristic of many insects (see above). The evasive reactions observed in tethered flight are much slower and only reflect to some extent what happens under free flight conditions (Tammero and Dickinson 2002; Bender and Dickinson 2006). Independent of these dynamic aspects, the studies agree that asymmetries in the OF pattern on both eyes, which is largely translational during intersaccadic intervals, along with a strong looming component, are crucial for controlling the direction and amplitude of saccades leading to collision avoidance. However, it is not yet clear which of the various parameters that characterise the asymmetries in binocular OF are most important. The asymmetry of the flow pattern may be due to the location of the expansion focus in front of one eye or to a difference between the total OF in the visual fields of the left and right eye (Bertrand et al. 2015; Serres and Ruffier 2017; Thoma et al. 2020). In this context, bumblebees were shown to extract the maximum rate of image motion in the frontal visual field and in this way steer away from obstacles (Lecoeur et al. 2019). Thereby, the bees’ maximum evasive acceleration depends linearly on the relative retinal expansion velocity of the obstacle, i.e., the ratio of the retinal image expansion of the obstacle to its retinal size (Ravi et al. 2022). Not the whole visual field appears to be involved in controlling saccadic turns that lead to collision avoidance manoeuvres. In blowflies, for example, OF in the lateral visual field plays no role in determining the direction of avoidance saccades. This property is probably related to the flight style of blowflies. During intersaccadic intervals, they fly predominantly forward, with some lateral components immediately after the saccades that shift the expansion pole of the OF slightly towards the frontolateral eye regions (Kern et al. 2012). In contrast, *Drosophila*, hoverflies, but also bees can hover and fly sideways to a certain degree. Here, lateral and even posterior parts of the eye can get involved in triggering evasive saccades as part of a collision avoidance flight strategy (Braun et al. 2012; Geurten et al. 2012; Muijres et al. 2014).

### Negotiating gaps in cluttered environments

**Fig. 5:**
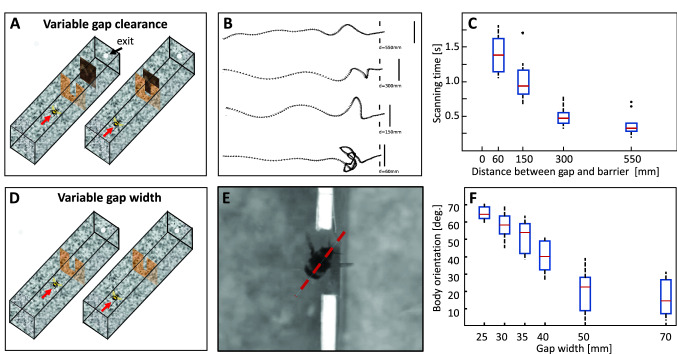
Gap negotiation. Bumblebees are able to relate the width and clearance of a gap to their body-size when they want to pass it. The experimental analysis was done in flight tunnels with an obstructing wall either containing a gap with a barrier behind it in a variable distance (**A**) or a gap of variable width (**D**). In either constellation bumblebees decelerate at some distance to the wall with the gap and approached the gap with increasing lateral displacements depending on distance of the barrier to the gap (**B**) or the width of the gap (not shown). The duration of these lateral scanning manoeuvres containing motion parallax information increase with decreasing distance between the gap and the barrier (**C**). The bees are likely to use this parallax information to assess the distance between gap and barrier and/or whether the gap was sufficiently wide to be able to fly through headfirst in normal flight orientation or whether it had to pass in an oblique orientation (**E**). Because the wingspan is larger than the body length, the bumblebees flew almost sideways through narrow gaps (**F**) (Data from Ravi et al. 2019, 2020)

In cluttered natural environments, insects not only have to avoid obstacles. Often, they also must fly through small gaps without damaging their wings. To do this, they must assess whether a gap itself and the clearance behind the gap are large enough to fly through it unharmed. The assessment of the passability of gaps is an elementary task of spatial vision and aerial flight control. Whereas a brightness-based strategy for gap detection and negotiation was concluded to be a fast, computationally simple and efficient mechanism for orchid bees living in tropical rainforests (Baird and Dacke 2016), bumblebees have been shown to employ a specific active vision strategy to generate the OF required to assess the passability of a gap and to ensure injury-free passage. When bumblebees approach a gap, they already slow down at some distance if the clearance behind the gap or the width of the gap appear to fall below a critical size (Fig. 5A, D). A rough estimation of these critical measures thus seems to be possible already in the normal cruising flight mode through the translational OF during the intersaccades. While the bumblebees reduce their forward speed, they increase their sideways speed and perform mainly sideways scanning movements in front of the gap (Fig. 5B) providing OF-based spatial information essentially in the frontal visual field. As the clearance behind the gap or the width of the gap decreases, bumblebees spend more and more time on these scanning movements (Fig. 5C). Based on the distinct translational OF in the frontal visual field, bumblebees appear to evaluate the geometry of the gap and thus its passability (Ravi et al. 2019). Through this active flight strategy, bumblebees are able to assess whether a gap is wide enough to fly through head-first in the normal direction of flight, or whether they need to pass at an angle or even sideways (Fig. 5E, F). The bees thus seem to "know" their own body size along the different axes, because they behave in this peculiar way even if they had no previous experience with the respective environment. Recently, it could be shown that this elaborate gap crossing behaviour scales with the body size of the animals; body size and wingspan can vary by up to a factor of two even within a given colony: small bumblebees show the characteristic scanning behaviour and typical sideways passages only at smaller gaps than do larger animals (Ravi et al. 2020). Hence, flying bumblebees perceive the affordance of their surroundings relative to their individual body size to navigate safely through complex environments.

### Optic flow in local navigation based on landmark information

All flying insects should be able to avoid collisions with obstacles and to negotiate narrow gaps in three-dimensional environments, but also to interrupt a flight by landing on an object. In contrast, navigation between the nest and food sources is a special ability of Hymenopterans such as bees, some wasps and ants, which take care of their brood and therefore need to return to their nest after foraging (Zeil et al. 2009; Zeil 2012; Collett et al. 2013; Zeil 2022). Basic components of navigation, in particular place memory, i.e., learning a location in space using visual cues in its vicinity, could also be identified in walking *Drosophila* (Ofstad et al. 2011). Visual landmarks represent important spatial cues and are used to locate an often barely visible goal, such as the nest hole in solitary bees or bumblebees. Information about the landmark constellation around the goal location is gained in characteristic learning flights during which the animal frequently orients towards the goal location or objects in its vicinity (Lehrer 1991; Zeil 1993a, b; Hempel de Ibarra et al. 2009; Philippides et al. 2013; Riabinina et al. 2014; Lobecke et al. 2018). During these learning flights, the animals are thought to gather relevant information about the landmark constellation, which can then be used to localise the goal when returning to it after an excursion. Although a variety of visual features such as contrast, texture and colour are relevant for defining landmarks and are used to locate the goal (Collett and Collett 2002; Zeil et al. 2009; Zeil 2022), there is evidence that the spatial arrangement of the landmark constellation can also play an important role and that landmarks defined solely by motion cues can be used for goal location (Lehrer and Collett 1994; Dittmar et al. 2010, 2011). Although the mechanisms by which landmark constellations are learned and what information is eventually used to locate the goal are not yet fully understood, the OF information actively generated during typical learning and search flights of bees is essential for the acquisition of spatial memory of the goal environment (Zeil et al. 2009; Zeil 2012). Moreover, in the proximity of landmarks, animals adjust their flight movements depending on the landmarks’ specific textural properties. Landmarks near the goal are best suited for its localisation because the retinal positions of near landmarks are shifted more during translational movements than those of more distant ones. Hence, special weight is implicitly given to nearby landmarks when navigating close to the goal—just because of the geometric properties of the OF during intersaccadic translational locomotion. The processing of behaviourally relevant visual information is thus facilitated by the characteristic active gaze strategy in free flight (Egelhaaf et al. 2012).

### Estimation of flight distances: path integration in flying insects

**Fig. 6:**
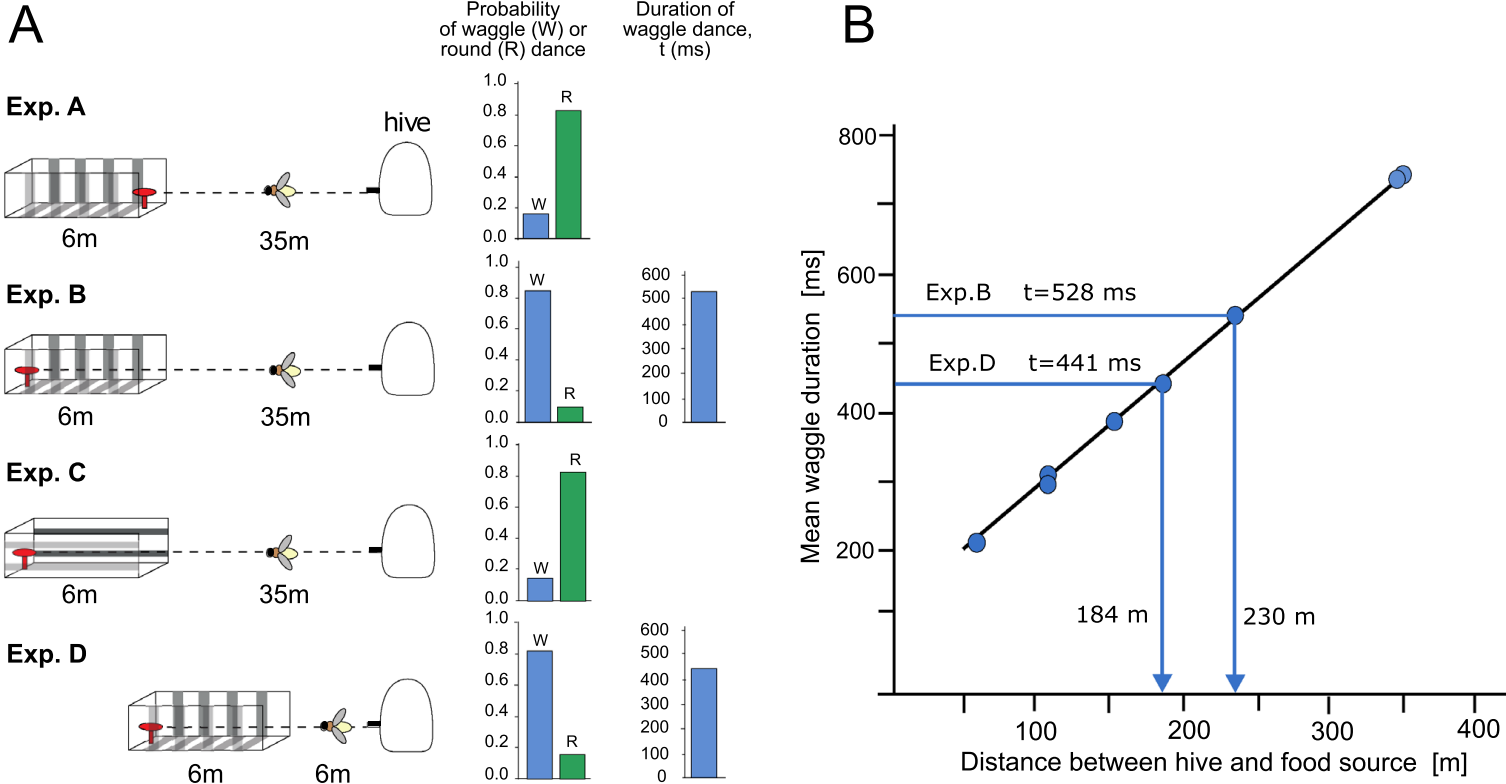
Estimation of flight distances: Path integration in bees. Honeybees measure distances in terms of OF generated during flight and communicate this information to their hive mates by the waggle dance. Behavioural analysis revealed how honeybees estimate the distance between their hive and a food source. **A** Experimental layout for experiments using flight tunnels (left diagrams) and probabilities with which a round dance (R; green bars) or a waggle dance (W; blue bars) was performed by the bees in the respective situations and, if applicable, duration of the waggle dance, with which the distance between food source and nest perceived by the bee is indicated (right diagrams). The walls of the tunnel were either covered with a texture that contained vertically oriented (Exp. A, Exp. B, Exp. D) or horizontally aligned stripes (Exp. C). The bees were trained to collect sugar water from a food source (indicated by red object). When the food source was placed at the entrance of the tunnel (Exp. A), the bees performed mainly round dances after returning to their hive, signalling a short distance to the food source. When the food source was placed at the end of the tunnel containing vertically oriented texture (Exp. B), the returning bees performed mainly waggle dances, signalling much larger distances to the hive, although the actual travel distance was not much larger. A food source at the same distance, however, located in a tunnel with horizontally oriented stripes (Exp. C), again led mainly to round dances. The main difference between Exp. B and Exp. C is that in the former much OF is evoked on the eyes of the bee while flying along the tunnel, whereas in the latter case, there is only little OF, because the contours are oriented along the flight direction. When the tunnel covered with vertical contours and the food source close to its end is placed near to the hive (Exp. D), mainly waggle dances are performed, which are shorter than those performed in Exp. B (compare blue bars). These experiments suggest that travelled distance is measured in terms of OF. **B** Calibration of the odometer of the bee. Mean duration of waggle dances elicited by outdoor feeders at various distances to the hive. Also shown are the mean durations of waggle dances measured in Exp. B and Exp. D and their equivalent outdoor flight distances, as read from the regression line. These findings show that OF-based distance measurements e.g., in the context of path integration depend much on the spatial layout of the environment and are thus highly ambiguous (Adapted from Srinivasan et al. 2000)

OF information is used at least by flying Hymenoptera (honeybees, bumblebees; solitary wasps), but also to a certain extent by ants, for spatial tasks related to navigation over long distances from their nest site. They monitor travel distances and directions when foraging on often curvy and complex routes in an unknown environment to be able to return to their nest later. While the direction of locomotion is determined by employing compass information provided by the sun and the associated polarisation pattern in the sky and/or by other directional cues (Homberg 2004; Pfeiffer and Homberg 2007; Wolf 2011; Seelig and Jayaraman 2015; Srinivasan 2015; Heinze et al. 2018; Wehner 2020), OF experienced during flight to a food source or some other behaviourally relevant location, such as the nest, plays a crucial role in determining the distance travelled; OF in both the lateral and ventral visual field, depending on the spatial layout of the surroundings, is used for estimating the distance travelled (Chittka et al. 1995; Esch and Burns 1996; Srinivasan et al. 1996, 1997, 2000; Esch et al. 2001; Hrncir et al. 2003; Tautz et al. 2004; Srinivasan 2014). The measured directions and the corresponding distance estimates are combined to determine the vector for the direct route back to the goal location (‘path integration’). However, the OF generated by translatory locomotion depends on both the flight speed and the spatial layout of the environment flown through, i.e., in particular the distance to objects in the surroundings and—in the ventral visual field—to the ground (see above). This means that even for a given flight velocity the OF and, thus, the distance estimates based on it are highly ambiguous. It all becomes even more demanding regarding estimates of distances travelled, as both the lateral and ventral optic flow are not only used to assess the distances travelled, but also for regulating flight velocity and/flight height (Baird et al. 2005, 2006; Portelli et al. 2010, 2017; Linander et al. 2015). Behavioural experiments with honeybees in both experimenter-designed and natural environments revealed that these ambiguities are both reflected by the characteristics of the waggle dances, with which honeybee foragers communicate to their hive mates the estimated direction and distance to prolific food sources, but also the locations where the recruited bees should search for food after manipulating the environment in a dedicated way (Fig. 6) (Srinivasan et al. 2000; Esch et al. 2001; Tautz et al. 2004). These environment-dependent ambiguities of OF-based estimates of distances might be a serious challenge to finding a goal location using path integration unless certain conditions are met: (1) The flight altitude, independent of how it is controlled (see above), should be quite similar on the outbound and return flight. (2) The environment in which the outbound flight takes place and during which the direction and distance from the starting point are determined by path integration, should not differ statistically in terms of its spatial layout (e.g., density and distance of environmental objects) from the environment in which the animal flies back as straight as possible according to the path integration vector. (3) The perceived OF depends at any time on the current distance and texture to the objects in both the lateral and ventral visual field, even after some spatial pooling. Thus, the OF-based distance measurements especially in cluttered terrain fluctuate in time. To smooth out these fluctuations and to obtain a reasonably accurate estimate of the distances travelled, integration of the OF over a sufficiently long time period is necessary (Meyer et al. 2011; Schwegmann et al. 2014b). This means that path integration relying on OF-based measurements of travel distances are reasonably accurate only on a relatively large spatial scale, whereby this spatial scale is expected to depend on the statistic properties of the spatial layout of the environment. This might not be too much of a problem for flying hymenopterans, if they do not only rely on path integration, but can also use previously learned landmarks in the environment to find their goal again.

## Optic flow computation, representation of spatial information in the insect brain and behavioural control

**Fig. 7:**
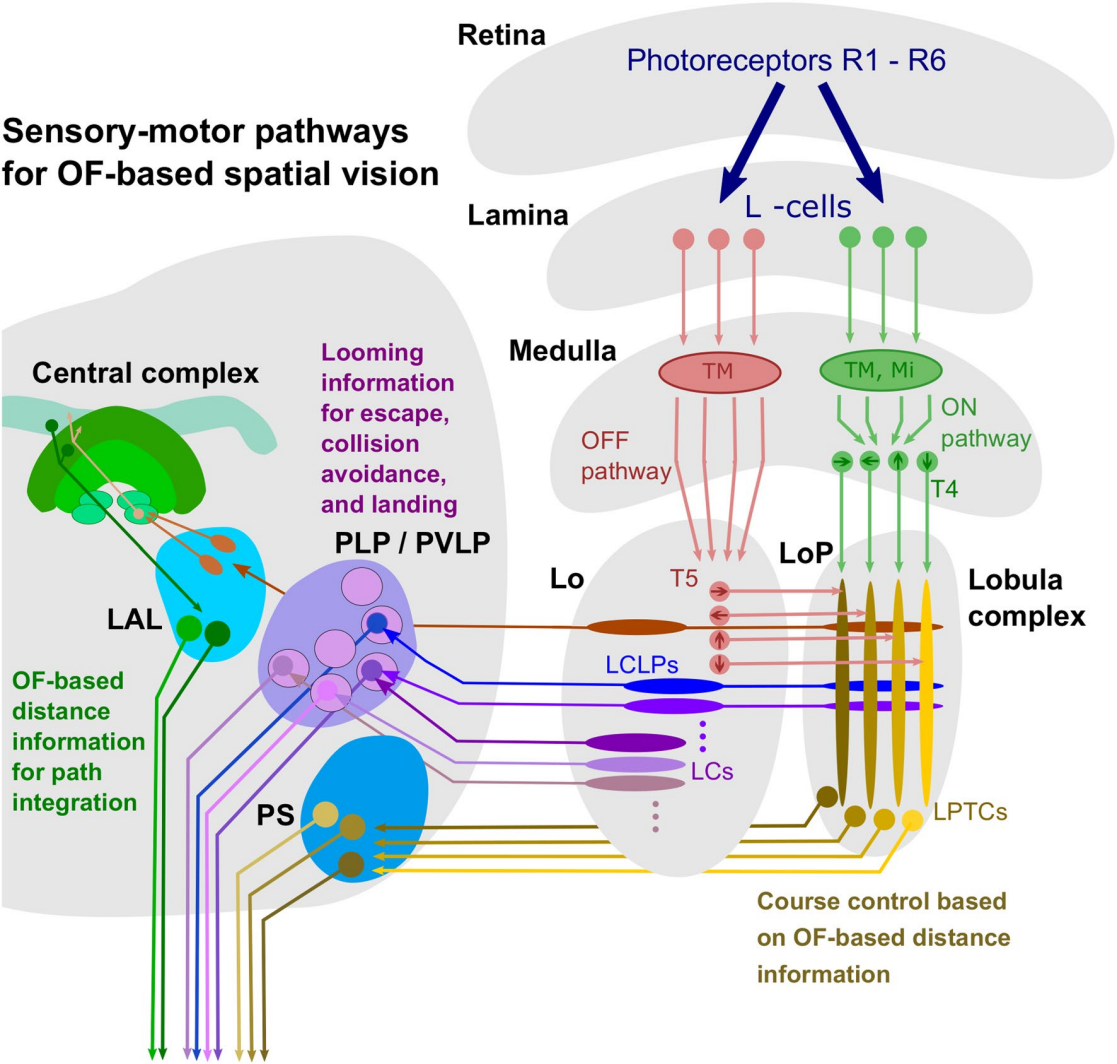
Optic flow processing in insect nervous system. Schematic of the visual motion pathways involved in the processing of OF-based spatial information. Shown is only one brain hemisphere including the central complex located centrally in the protocerebrum. The scheme is largely based on the visual pathway of flies, even though individual elements have been adopted from studies on other insects. The changes in brightness induced by motion signals are perceived in the retina by the retinotopic array of photoreceptors R1–R6 and then temporally filtered in the first visual neuropil, the lamina, by different types of L-cells. Thereby the visual information is divided into an ON (red) and OFF channel (green). Local motion detection takes place in the ON and OFF channels in networks of local retinotopic interneurons (Tm, Ti) in the medulla. The retinotopically organised outputs of the motion detection networks are formed by the T4 (ON channel) and T5 cells (OFF channel), which receive their input signals in the medulla (T4 cells) or in the anterior part of the lobula complex, the lobula (T5 cells). At the level of the lobula complex, the different pathways for OF-based spatial vision segregate at least partially. In the lobula plate, retinotopic motion information mediated by the T4 and T5 cells is spatially pooled by the lobula plate tangential cells (LPTCs), which, with their large receptive fields, provide global OF information as induced by the animal's own motion. Part of these cells are connected to descending neurons in the posterior slope region (PS) in the protocerebrum; these descending neurons are involved in mediating a wide range of components of course control in three-dimensional environments. Movement information, but also other visual information, is transmitted in the lobula to the retinotopically organised lobula columnar (LC) cells and, if applicable, additionally in the lobula plate to the lobula plate—lobula columnar (LCLP) cells. Some of these cells act as projection neurons, transmitting looming information to the optic glomeruli of the posterior lateral protocerebrum (PLP) and posterior ventrolateral protocerebrum (PLVP), from where this information is transmitted via dedicated descending neurons to control escape as well as landing behaviour. OF information required for distance measurements in the context of OF-based path integration is conveyed from the lobula complex into the lateral accessory lobe (LAL), from where it is fed via the noduli into the intricate neuronal circuits of the central complex. The information about the integrated path distance, suitably combined with directional information, is transmitted via the LAL and descending neurons to control navigational behaviour

The retinal OF patterns, defined as shifts in the geometric projections of objects in the environment on the retina induced by the animal's own movements (Koenderink 1986; Strübbe et al. 2015), are not directly available to the visual system. Rather, the input of the array of photoreceptors is provided by the time-dependent brightness changes evoked during self-motion; these are transformed into electrical photoreceptor signals for further processing in the subsequent neuropil layers of the optic lobes, i.e., the lamina, the medulla, and the lobula complex, which is subdivided in flies into the lobula, and lobula plate (Fig. 7). OF information is initially determined locally by a large number of motion detectors that are arranged retinotopically; they jointly subserve the entire visual field. This local motion information and the spatial information it contains may then be combined with other visual information and further processed in partly parallel pathways involved in mediating the different components of spatial behaviour. These pathways are supplied by visual projection neurons that carry information from the optic lobes to distinct regions of the central brain and eventually via descending neurons to the motor control centres in the thoracic ganglia (Fig. 7).

### Local optic flow processing in early visual areas

The neural circuits of motion detection receive their input from a retinotopic array of discrete sampling points of the ommatidial lattice. The relatively low spatial resolution of insect eyes does not seem to be a disadvantage, as many insects are quite capable of performing highly aerobatic flight manoeuvres and solving challenging spatial tasks based on OF information (see above). The computational mechanism that has been proposed as the basis for local visual motion processing in flying insects is the correlation-type motion detector (Reichardt 1961; Borst and Egelhaaf 1989, 1993; Egelhaaf and Borst 1993). In its simplest form, a local motion detector consists of two mirror-symmetric subunits. In each subunit, the spatially and temporally filtered brightness signals from neighbouring points in visual space are combined by a multiplicative interaction after one of them has been delayed. The final response of the detector results from the subtraction of the output signals of two such subunits with opposite preferred directions, greatly improving the directional selectivity of the motion detection circuit. Each motion detector responds with a positive signal to motion in a particular direction, i.e., either horizontally or vertically, and with a negative signal to motion in the opposite direction. Different variants of this basic motion detection scheme have been proposed to explain the responses of insect motion-sensitive neurons under a variety of stimulus conditions, including even natural OF as experienced under free-flight conditions (Borst and Egelhaaf 1989; Franceschini et al. 1989; Baird et al. 2013; Egelhaaf et al. 2014; Mauss et al. 2017; Borst 2018; Yang and Clandinin 2018; Chen et al. 2019; Zavatone-Veth et al. 2020; Kohn et al. 2021). By combining the sophisticated toolkit of genetic and molecular approaches in *Drosophila* with electrophysiological and imaging techniques, great progress has been made in recent years to identify the different cellular elements of the neural circuits underlying local motion detection. From a functional point of view, a particularly relevant result is the separation of the motion detector input circuits already at the level of the lamina into ON and OFF channels that processes brightness increases and decreases separately (Fig. 7). This separation is maintained in the intricate neural circuitry of motion detection; the ON and OF motion signals and the spatial information they carry are then fed retinotopically by two types of neurons (T4, T5) into the downstream processing of the OF. Because of the existing very good reviews on the cellular implementation of the local mechanisms of motion detection, this important aspect of OF computation will not be elaborated on here (Mauss et al. 2017; Strother et al. 2017; Yang and Clandinin 2018; Borst et al. 2020).

**Fig. 8:**
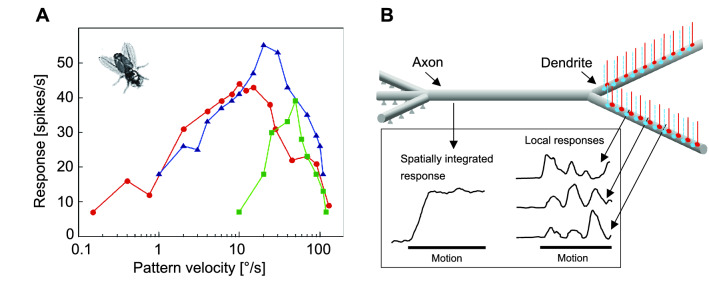
Local vs. spatially integrated optic flow representation. **A** Time averaged velocity responses of a wide-field neuron in the blowfly third visual neuropile, the lobula plate, to periodic stripe patterns of different spatial wavelengths (red curve: 6.6°; blue curve: 21.5°; green curve: 36.3°) moving horizontally in the neuron’s preferred direction. Velocity-response curves strongly depend on the pattern properties; their optima are shifted to higher velocities with increasing spatial wavelength of the pattern (Data from Eckert 1980). **B** Consequences of dendritic integration on the representation of visual motion: Schematic of a directionally selective wide-field neuron with two branches of its dendrite, the axon and the axon terminal. The wide-field neuron receives retinotopically organized input from many local motion sensitive elements (vertical lines terminating with synapses indicated by red dots (excitatory synapses) and blue dots (inhibitory synapses) on the dendrite). Because of this input, the cell is excited by motion in its preferred direction and inhibited by motion in its null direction. Even when the velocity of motion is constant, the activity induced by the local movement sensitive elements is modulated depending on the texture of the stimulus pattern within their respective receptive field. Traces on the right indicate the time-dependent signals of three local input elements of the wide-field neuron. By dendritic pooling of many local elements, this pattern dependence in the time course of the responses is reduced (left trace)

From a functional perspective, it is important to note that the local motion detectors do not provide a veridical representation of the OF—with several consequences for the OF-based spatial information: (1) *Velocity dependence*: Local motion detectors do not function like odometers, even if the temporal and/or spatial average of their responses is taken into account. Their average response amplitude increases with increasing speed, reaches a maximum and then decreases again; so biological movement detectors do not uniquely reflect the motion speed. The response characteristics of biological motion detection systems are even more complex, as their velocity maximum depends on the textural properties of a moving stimulus pattern, or transferred into a natural world, on the texture and shape of objects the animal flies by (Borst and Egelhaaf 1993; Egelhaaf and Borst 1993; Egelhaaf et al. 2014) (Fig. 8A). The pattern dependence of velocity tuning is less noticeable when the pattern consists of a broad spectrum of spatial frequencies (Dror et al. 2001), as is characteristic of natural scenes (Schwegmann et al. 2014b). Despite these ambiguities, free-flying flies and bees, those insects in which this important aspect has been particularly thoroughly studied, appear to regulate their translational velocities in such a way that retinal velocities remain within the part of the working range of the motion detection system where its response increases monotonically with velocity. (2) *Time course of local motion responses*: The responses of local motion detectors do not unambiguously reflect the local pattern speed, since they depend to a large extent on the local texture properties and the spatial layout of the visual environment. Since the response modulations of neighbouring motion detectors are out of phase with each other, spatial pooling of many such detectors reduces the pattern-dependent response modulations. There is thus a kind of trade-off between the spatial resolution with which the movement information is perceived and the quality of the represented time course of local pattern velocity (Fig. 8B) (Egelhaaf et al. 1989; Meyer et al. 2011; O'Carroll et al. 2011). (3) *Motion adaptation*: Motion-sensitive neurons adapt their response strength to the given stimulus conditions. This is inevitably at the expense of veridical velocity encoding, but improves sensitivity to velocity changes, such as those that may occur when flying past nearby objects. This property has been interpreted as an adaptation to facilitate the detection of spatial discontinuities (Liang et al. 2008, 2012; Kurtz 2012; Li et al. 2017). Motion adaptation occurs at multiple processing levels of the visual motion pathway, both at the level of local motion sensitive elements and at the level of downstream spatial integrating cells (see below). As a functional consequence of the direction-independent movement adaptation at the level of local movement detection, the representation of the local direction of movement is independent of the overall adaptation status (Li et al. 2021).

**Fig. 9:**
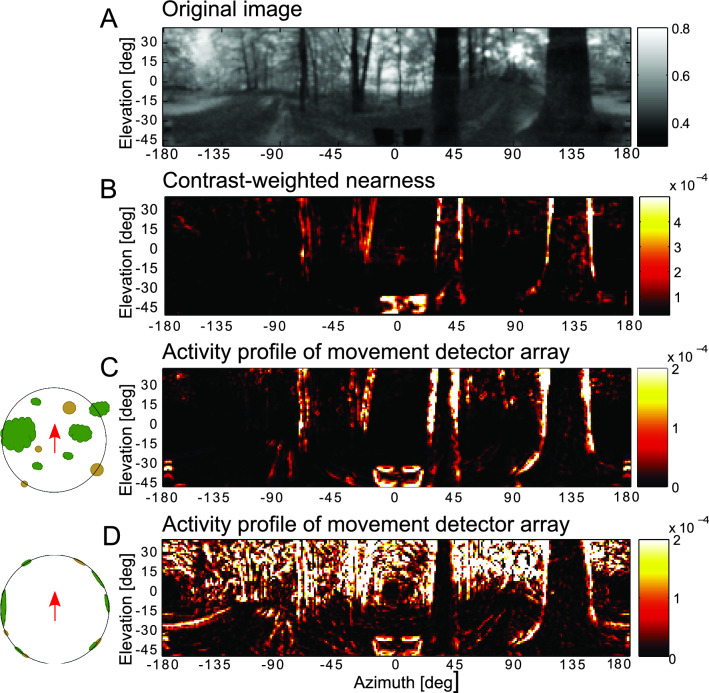
Local motion measurements represent contrast weighted nearness in natural environments. Encoding of spatial information by two-dimensional arrays of local movement detectors during translatory locomotion in a natural environment. All images represent one instant of time during a longer translation sequence. **A** Original input image (after a nonlinear Naka-Rushton-like transformation of brightness values). **B** Contrast-weighted nearness map: The nearness (i.e., the inverse distance of the observer to any point in the 3D environment) is multiplied by the local contrast at the corresponding image location, resulting in the largest contrast-weighted nearness values at the edges of nearby objects (colour code in arbitrary units). **C** Activity profile of the array of movement detectors while the observer moves through a natural environment staggered in depth (see inset). The activity profile is given by the absolute values of horizontally and vertically aligned local movement detectors; model simulations based on an elaborated version of the correlation-type movement detector (colour code arbitrary units). The activity profile reflects the contrast weighted nearness structure of the three-dimensional environment. **D** Activity profile of the array of movement detectors at one instant of translatory motion after the depth structure of the forest environment has been equalized by projecting it on the surface of a sphere (see inset). Model simulation as in **C**. The activity profile now reflects all contours in the environment irrespective of their distance (Data from Schwegmann et al. 2014a, b)

These characteristics of the local movement detectors have direct consequences for the spatial information that is represented at this processing stage and made available for further downstream processing. Since the retinal speed of an object scales inversely with distance during translational locomotion of the animal, a nearby object leads to stronger local movement detector responses than more distant objects. As a consequence, a visual scene is segregated into near and far objects without much computational effort, as demonstrated by computer modelling based on physiological data (Egelhaaf et al. 2014; Schwegmann et al. 2014a). Since motion responses are elicited only by textured surfaces or object boundaries, the array of local motion detectors represents with increasing response strength the proximity of object contours rather than the nearness to the objects themselves (Fig. 9). This representation of spatial information is further enhanced by the motion adaptation processes operating at the level of local movement detectors (Li et al. 2017).

### Optic flow processing for behavioural control in higher order brain regions

The local retinotopically organised motion signals computed in the early visual motion areas provide the input information of the downstream computational processes that are involved in mediating the different types of OF-based spatial behaviour. OF-based behaviour relies on global, not just local, characteristics of the OF fields that are actively induced on the eyes during locomotion. This means that the mechanisms mediating the different components of spatial behaviour must combine local motion measurements from different parts of the visual field and, if necessary, bring them together with information from other visual channels depending on the requirements of the spatial task. This is done in parallel pathways depending on the respective behavioural tasks and the spatial information required. Three such pathways, all involved in solving OF-based spatial vision tasks, will be briefly outlined here (Fig. 7).

#### Global optic flow sensing and its dependence on the spatial structure of the environment

Much is known about the integration of local motion information over large parts of the visual field to estimate the different components of the animal's self-motion. This is relevant for course stabilisation in space, but also in other behavioural contexts, such as the regulation of flight speed depending on the spatial structure of cluttered environments (see above). The underlying mechanisms of OF processing have been studied in detail in flies. Pooling of local motion information takes place in the lobula plate on the extended dendrites of wide-field neurons, the lobula plate tangential cells (LPTCs). The different LPTCs respond in a characteristic manner to the direction of the animal's self-motion depending on their respective input organisation. The specificity for the different types of OF patterns is further enhanced by synaptic interactions between LPTCs within the same and/or both brain hemispheres (Hausen 1984; Krapp 2000, 2014; Egelhaaf and Kern 2002; Egelhaaf 2006; Borst et al. 2010; Hennig et al. 2011; Egelhaaf et al. 2012; Liang et al. 2012; Borst 2014; Hardcastle and Krapp 2016; Mauss and Borst 2019). Apart from providing information about self-motion of the animal, these neurons also represent during translatory self-motion OF-based spatial information as averaged within the confines of the cells’ receptive fields (Kern et al. 2005; Liang et al. 2012; Ullrich et al. 2015). The neuronal representation of spatial information is further enhanced by motion adaptation (see above; Liang et al. 2008; Li et al. 2017). The retinal velocity range, and thus the spatial range that can be encoded, is constrained by the inevitable neuronal noise resulting from the biophysical mechanisms of signal transduction in photoreceptors and cellular information processing (Laughlin 1994; Grewe et al. 2003, 2007; Warzecha et al. 2013). Under spatially constrained conditions, where flies fly at translation velocities of only slightly more than 0.5 m/s, the spatial range within which significant distance-dependent information is represented by LPTCs during intersaccades is about 2 m (Kern et al. 2005). Since retinal speed decreases with increasing distance to an object and increases with increasing translational velocity, a given retinal speed is achieved by different combinations of object distance and translational velocity. The behaviourally relevant spatial range thus scales with the intersaccadic translational velocity. From an ecological perspective, this scaling of the behaviourally relevant depth range is functional: a fast-flying animal should, for example, initiate a turn leading to an evasive manoeuvre or a deceleration when approaching an object to land at an earlier time and at a larger distance to the object than a slow-flying animal (Egelhaaf et al. 2012). This expectation matches, at least qualitatively, the characteristics of insect landing behaviour (see above). Furthermore, motion-sensitive wide-field neurons show a characteristic time-dependent response profile that reflects the spatial landmark constellation surrounding a behaviourally relevant goal of bees or bumblebees, such as the nest hole or a food source (Egelhaaf et al. 2014; Mertes et al. 2014). The information about rotational and translational self-motion is conveyed by these wide-field neurons to the posterior slope region of the central brain and from here via descending neurons to the motor control centres in the thoracic ganglia, but also to muscles that are responsible for the animal's head movements and thus head–body coordination (Fig. 7). The information conveyed by these descending neurons is assumed to play a role in stabilising an intended course of the animal against involuntary disturbances, in coordinating head and body movements, e.g., keeping the head horizontally aligned while the body needs to make roll movements during sharp curves (‘banked turns’) and during sidewards movements (Haag et al. 2007; Huston and Krapp 2008; Wertz et al. 2008, 2009, 2012; Suver et al. 2016; Namiki et al. 2018). During intersaccadic translatory self-motion, these pathways may also provide the global nearness information required for velocity control or balancing the flight trajectories in densely cluttered environments (see above).

#### Processing of looming OF for escape, collision avoidance and landing

When an animal approaches an object, such as a landing site or an obstacle, the OF pattern on the eyes expands (‘looming’). Depending on the behavioural state of the animal, the situational context, and the dynamics of the looming stimulus, different behavioural responses may be appropriate, i.e., either an escape response, an avoidance response, or a landing response. Expanding OF fields can, in principle, be encoded by integrating the output signals of several local motion-sensitive elements with suitable preferred directions in specific areas of the visual field. Evidence for a functional role of such local motion sensitive elements in encoding looming stimuli comes from experiments where the extension of the legs, as is characteristic of landing reactions, and evasive behaviour of tethered-flying *Drosophilae* in response to looming stimuli could be eliminated by opto-genetically switching off such local motion-sensitive elements (Schilling and Borst 2015). In flies, a population of visual projection neurons in the lobula, the lobula columnar (LC) cells (Fig. 7), plays a key role in mediating these and other behavioural responses, as could be shown by optogenetic activation experiments (Wu et al. 2016). The LC neurons can be classified into distinct anatomical types with response specificities for different visual features. Each LC type comprises several retinotopically arranged neurons with similar morphology, whose individual dendritic branches extend over part of the lobula column arrangement and, in some of these neurons, also over part of the lobula plate (lobula plate—lobula columnar (LPLC) cells; Fig. 7). Hallmark of most of these neurons is the convergence of their axons onto cell-type specific target regions in the central brain, often referred to as ‘optic glomeruli’ (Panser et al. 2016; Wu et al. 2016; Timaeus et al. 2020; Klapoetke et al. 2022). Looming stimuli are detected by specific types of LC as well as LPLC cells that distribute as projection neurons looming information from the optic lobe to several of the nearly 20 optic glomeruli in the posterior lateral protocerebrum (PLP) and posterior ventrolateral protocerebrum (PLVP) (Fig. 7). A pathway transferring visual looming information from these optic glomeruli via a giant descending neuron to the motor control centres in the thoracic ganglia elicits looming-induced escape responses (Fig. 7) (von Reyn et al. 2014; Klapoetke et al. 2017, 2022; Ache et al. 2019a, 2019b). Further descending neurons that mediate looming stimulus-induced landing receive their visual input from other types of visual projection neurons that combine input from the lobula and lobula plate. These descending neurons are gated by the behavioural state of the animal, i.e., their gain is severely attenuated if the animal does not fly and, thus, the initiation of landing would be inappropriate (Ache et al. 2019a). Thus, gating of looming responses of these descending neurons is a mechanism that ensures a meaningful context-dependence of the control of spatial behaviour.

#### Translational OF processing for path integration

The spatial behaviours and their respective neural basis considered so far are involved in solving tasks on a time scale of a few milliseconds to a few tens or hundreds of milliseconds at most. Path integration in the context of navigation behaviour takes place on a much longer time scale. Here, the translational OF components and the corresponding directions of locomotion during excursions to a target, e.g., a food source (see above), have to be integrated over times of several seconds up to the minute range. In recent years, essential aspects of OF-based estimation of travel distances have been elucidated, which could play a key role in path integration. To provide a neural representation of travel distance based on OF, the activity of motion-sensitive cells in appropriate areas of the visual field must be integrated over relatively long periods of time, taking into account the respective direction in which the animal was moving. Thus, path integration implies that direction and distance information are continually combined in an appropriate way. One brain region, the central complex, has emerged as the likely site of path integration in the insect brain, where the necessary distance and directional information is computed and brought together. The different areas of the central complex with their regular, repetitive neuroarchitecture with 16–18 vertical columns and several horizontal layers could be characterized anatomically, electrophysiologically and with imaging approaches as a functionally ring-shaped computational system and modelled based on the experimental data (Pfeiffer and Homberg 2014; Turner-Evans and Jayaraman 2016; Webb and Wystrach 2016; Varga et al. 2017; Heinze et al. 2018; Honkanen et al. 2019; Webb 2019; Pabst et al. 2022). Representations of directional information that can be used as a compass in path integration are found in this structure in a wide range of insects. Depending on the species and the specific ecological conditions under which they navigate, this information comes from different sources of information, such as in particular the celestial compass provided by the sun and/or the polarisation pattern of the sky. Directional information can, however, also be derived from distant landmarks or by integrating estimates of rotational movements of the animal based on the rotational OF and/or proprioceptive signals to encode the current course of the insect (Heinze and Homberg 2007; Seelig and Jayaraman 2015; Turner-Evans et al. 2017; Green and Maimon 2018; Rosner et al. 2019; Pisokas et al. 2020). Path integration requires combining directional signals with information about the distances travelled. As mentioned above, bees use translational OF to estimate travel distances, while walking animals like ants primarily rely on integrating their steps (Collett and Collett 2017; Stone et al. 2017; Ronacher 2020). In several insect groups the central complex could be shown to house neurons sensitive to wide-field motion (Bausenwein et al. 1994; Kathman et al. 2014; Weir et al. 2014). In bees, a set of four prominent neurons in the noduli of the central complex could be characterized that respond to translational OF in a speed-dependent manner. They respond best to backward or forward flight in directions that deviate by about 45° from the animal's central midline (Stone et al. 2017). These neurons thus divide the animal's movement space into four cardinal directions and together can robustly encode all of the animal's translational movements—even if the body axis is not aligned with the direction of movement, e.g., when the animal makes lateral movements before crossing a narrow gap (see above). Although it is not yet understood in detail at the neural level how OF-based distance information is integrated in the context of path integration, possible mechanisms have been made plausible by several computational modelling approaches based on established connections between existing central complex neurons (Stone et al. 2017; Pabst et al. 2022). The cellular mechanism in effect leads to an accumulation of neuronal activity in each of eight directions represented by the population of direction cells. Since each accumulator unit acts as a separate directional odometer, there is no need for an overarching odometer cell representing the total distance travelled. Rather, the combined activity of all integrators represents as a distributed neural code for the home vector the distance to the starting point in relation to its direction at a given time during foraging (Stone et al. 2017).

## Conclusions

In flying insects, the OF generated on the eyes during locomotion is the main source of spatial information. Although other sources of spatial information, such as disparities in the two retinal images that form the basis for stereoscopic vision, are relevant for some insects (see above), stereoscopic vision can only be used in the immediate near field of the animal. However, especially during fast locomotion, behavioural decisions are necessary at much larger distances to behaviourally relevant structures in the respective environment.

Distance information, however, is only contained in the translational components of the OF; if these are overlaid by too strong rotational flow, it becomes computationally difficult to extract valid spatial information from the OF patterns. The saccadic flight and gaze strategy of many insects, in which changes in flight direction are squeezed into rapid saccadic turns and the animal moves essentially translationally during intersaccadic phases, is therefore interpreted as an active vision strategy that facilitates the evaluation of spatial information. This general flight and gaze strategy can be further refined (e.g., movement parallax or pivoting parallax) in specific behavioural contexts, e.g., when it is necessary to traverse narrow gaps or to determine distances of objects relative to a behaviourally relevant target near the animal.

However, OF can only be detected if there are contrasts or spatial discontinuities at object boundaries in the scenery. Accordingly, the array of retinotopically organised motion detectors in the early visual system does not provide a nearness map of the environment, but rather a contrast-weighted nearness map, i.e., information about the proximity of contours. This OF-based distance information is not metric, but scales inversely with object distance and is positively scaled with the animal's translational velocity. This means that a given value of an OF measurement at a large locomotion velocity corresponds to a larger object distance than at a smaller locomotion velocity. From an ecological perspective, these characteristics of OF-based distance information should not be a major problem in solving most spatial tasks, since object-related behavioural responses at higher locomotion speeds should already be initiated at a larger object distance than at a smaller speed. This applies especially to collision avoidance or the initiation of landings. An additional challenge arises if distance information based on OF is not determined directly to objects in the field of view, but if the distances over which the animal travels must be estimated, as is required for path integration between behaviourally relevant locations: Since the OF-based measurements of travel distances that are to be integrated depend on the spatial layout of the environment that the animal sees along its path as well as on its flight altitude, OF-based path integration can only lead to valid results if the animal flies at roughly the same height during its outbound and return flights and if the environment in which path integration takes place corresponds, at least statistically, in its spatial layout to the environment in which the animal eventually moves back to its starting point guided by the return vector determined in this way.

Overall, much progress has been made in recent years in understanding the mechanisms underlying OF-based spatial vision in different behavioural contexts, both at the behavioural and at the neural level. The neuronal processes of local motion detection could already be elucidated at a very high level of detail. This also applies to a large extent to the functional pathways for various aspects of spatial vision based on these local motion measurements, even though many questions are still open here. Moreover, it is still an exciting challenge to look at the detailed knowledge of the neuronal mechanisms of OF-based spatial behaviour not only from the point of view of cross-species commonalities of neuronal mechanisms, but also from the perspective of the species-specific adaptations of such mechanisms to the particularly interesting and often extreme behavioural feats performed, for example, by migratory locusts and monarch butterflies, by navigating Hymenoptera and several other fast-flying insect species in their rapid and usually collision-free traversal of dense, cluttered environments.

## Data Availability

This review article is based on a synopsis and interpretation of numerous previously published papers. No experimental data were obtained specifically for this review article.
